# Fly Fam161 is an essential centriole and cilium transition zone protein with unique and diverse cell type-specific localizations

**DOI:** 10.1098/rsob.240036

**Published:** 2024-09-11

**Authors:** Ankit Jaiswal, Andrew Boring, Avik Mukherjee, Tomer Avidor-Reiss

**Affiliations:** ^1^ Department of Biological Sciences, University of Toledo, Toledo, OH 43606, USA; ^2^ Department of Urology, College of Medicine and Life Sciences, University of Toledo, Toledo, OH 43614, USA

**Keywords:** centriole, centrosome, cilia, neuron, FAM161A

## Abstract

Family with sequence similarity 161 (Fam161) is an ancient family of microtubule-binding proteins located at the centriole and cilium transition zone (TZ) lumen that exhibit rapid evolution in mice. However, their adaptive role is unclear. Here, we used flies to gain insight into their cell type-specific adaptations. Fam161 is the sole orthologue of FAM161A and FAM161B found in flies. Mutating Fam161 results in reduced male reproduction and abnormal geotaxis behaviour. Fam161 localizes to sensory neuron centrioles and their specialized TZ (the connecting cilium) in a cell type-specific manner, sometimes labelling only the centrioles, sometimes labelling the centrioles and cilium TZ and sometimes labelling the TZ with varying lengths that are longer than other TZ proteins, defining a new ciliary compartment, the extra distal TZ. These findings suggest that Fam161 is an essential centriole and TZ protein with a unique cell type-specific localization in fruit flies that can produce cell type-specific adaptations.

## 1. Introduction

Centrioles are evolutionarily conserved subcellular structures that form the cells’ centrosomes and cilia and are essential for animal development and physiology (reviewed in [1]). Most animal cell types have precisely two centrioles; the older one usually forms a cilium (aka flagellum; reviewed in [2]). To form a cilium, the centriole attaches to the cell membrane, and its microtubules extend to form, first, a specialized segment known as the transition zone (TZ) and, later, through continued elongation, the main ciliary axoneme (reviewed in [2,3]). Various cell types have centrioles, TZs and axonemes with diverse morphologies, structures and functions [4]. For example, most centrioles, TZs and axonemes have ninefold symmetry; however, many sperm cells and some early embryonic cells have centrioles lacking ninefold symmetry (reviewed in [5,6]). Our understanding of the basic molecular mechanism that forms the centriole and basic organization of the cilium has grown extensively in the last 25 years, particularly in dividing somatic cells (reviewed in [7]). Yet, the molecular mechanisms underlying centriole and cilium cell type adaptation remain unclear.

In differentiated cells, centriole and TZ size and organization vary, reflecting cell type-specialized functions and animal biology (reviewed in [8]). This diversity is created by a conserved set of proteins that undergo a variety of adaptations [9]. These adaptations include gene duplication [10], gene loss ([9]; reviewed in [11]), new isoform splicing [12] and protein primary structural changes [13]. For example, in murine lineages, the primary structure of ‘family with sequence similarity 161 member A’ (FAM161A) evolved rapidly, resulting in the formation of a new isoform with distinct localizations and functions [13]. However, how these distinct centriolar variations are produced across different cell lineages is not completely understood.

One way to address this question is to use *Drosophila melanogaster*, a model animal with various centriole and cilium types (reviewed in [14]). A localization study of 14 centriole and TZ proteins across four sensory and sperm cell types by Jana *et al*. [4] found that cell type-specific localization of these proteins underlies diversification in the ultrastructure [4]. This careful analysis also found that the sensory TZ was divided into a proximal segment that contains most TZ proteins and a distal TZ segment that contains Cep290. However, the fly orthologue of FAM161A was not studied at that time.

FAM161A belongs to an ancient family of conserved centriole/TZ luminal proteins that are represented in humans by two paralogues (FAM161A and FAM161B) [15] and in worms by one orthologue [16]. In most mouse and human cells, FAM161A localizes to the centrioles [17,18]; however, in sensory neurons, such as mouse photoreceptors or ciliated neurons in worms, it also localized to the TZ lumen [16,19]. Its localization in flies is unknown.

Despite its presence in various tissues and cell types, the FAM161A mutation in humans has a narrow phenotypic spectrum, causing varying levels of retinitis pigmentosa corresponding to the severity of the mutation. The majority of cases are caused by a mutation in exon 3 coding a part of the UPF0564 domain [15,20,21], while some cases are caused by a mutation in exon 4 or 5 coding the end of the UPF0564 domain or the initial part of the C-terminal domain, respectively [22,23]. Outside of humans, FAM161A mutation phenotypes have been studied in only murine and canine models, in which, similar to humans, vision loss results from the mutational burden [24–26]. Non-mammalian FAM161 orthologues have not been studied functionally to date.

FAM161A in humans and mice is a centriole and TZ luminal scaffold component [18,19]. In the human centriole, this scaffold includes the proteins POC1B, POC5, Centrin 2, WDR90 and CCDC15 [18,27,28]. In mice, a specialized TZ of photoreceptors known as the connecting cilium includes POC5, centrin and FAM161A [19]. Mutations in the proteins that interact with FAM161A result in diverse phenotypes, some of which present similarly to FAM161A mutations; for example, mutations in POC1B, Centrin 2 and Centrin 3 cause retina degeneration [29,30]. Mutations in other proteins, such as POC5, result in a more diverse disease spectrum including retinitis pigmentosa [31,32]. Yet, the common characteristics of these phenotypes are reduced centriole and TZ structural integrity. These phenotypes are also consistent with the hypothesis that FAM161A, POC1B, POC5, WDR90 and Centrins form a centriole luminal scaffold that provides mechanical strength to the centriole and maintains its structural organization [18]. These luminal scaffolds also extend with some of these proteins into the connecting cilium [19]. To what extent these proteins are conserved in the connecting cilium of various cell types is unclear.

Only a few of the *centriole luminal scaffolds* have been studied in non-mammals. POC1 mutations in *Tetrahymena* and flies result in reduced centriole stability [12,33,34], a phenotype that suggests a centriole luminal scaffold. However, it is still being determined if fly Centrin has a similar function [35,36]. Flies do not appear to have clear orthologues for scaffolding proteins POC5, WDR90 and CCDC15 [9,37]. Finally, it is unknown if the fly centriole has a luminal scaffold and if fly luminal scaffold protein orthologues function as components of the TZ.

Here, we report that a single Fam161 family member exists in flies, acting as an essential centriole and TZ protein in sensory cells, exhibiting the greatest degree of cell type-specific localization in the connecting cilium of sensory neurons among scaffolding proteins with similar functions and even defining a novel domain: the extra distal TZ (found more distal to the distal TZ marked by Cep290). These observations suggest that Fam161 localization changes represent one mechanism for generating cell type-specific adaptation.

## 2. Results

### 2.1. Fly Fam161 is an orthologue of human FAM161A and FAM161B

**Figure 1.**
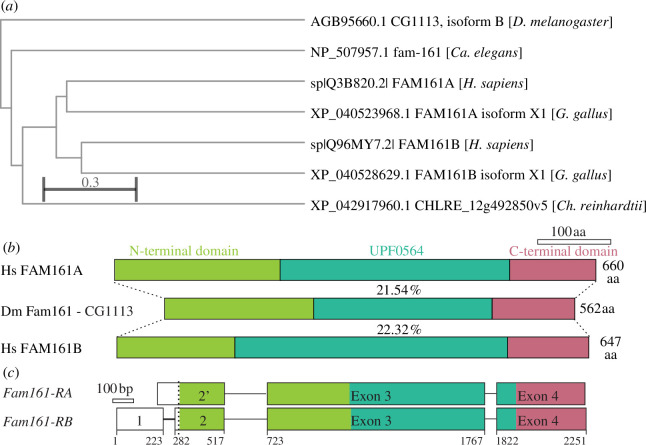
Fam161 (CG1113) is the co-orthologue of human FAM161A and FAM161B. (*a*) Phylogenetic tree of Fam161 family members generated using COBALT. The scale bar represents amino acid change per site. (*b*) Schematic representation of FAM161A (Q3B820), Fam161 and FAM161B (Q96MY7) proteins with a comparison of the N-terminal, central and C-terminal domains. (*c*) Predicted fly Fam161 transcripts (source: Ensemble).

FAM161A is a phylogenetically conserved gene with one paralogue, FAM161B, in vertebrates and one orthologue in invertebrates and protists [15,23]. To identify the fly Fam161 orthologue, we performed a reciprocal-blast-best-hit analysis using human FAM161A and the fly proteome. As was reported by Bandah-Rozenfeld *et al*. [23], this analysis did not yield an orthologue in the fruit fly genome [23]; however, a common FAM161A orthologue was found in insect species such as ants and butterflies. We then performed reciprocal blast analysis using monarch butterfly FAM161A and found that flies have one *fam161* gene, *CG1113*, that is orthologous to *Caenorhabditis elegans* Fam−161 and a co-orthologue of vertebrate FAM161A and FAM161B (figure 1*a,b*). Further reciprocal-blast-best-hit analysis also identified a FAM161 orthologue in *Chlamydomonas reinhardtii*: an uncharacterized protein CHLRE_12g492850v5. Interestingly, *Ch. reinhardtii* FAM161 is closer to human FAM161A and FAM161B than to fly Fam161, suggesting that invertebrate FAM161 has a more evolved primary structure. These data suggest that Fam161 is an ancient protein conserved from single-cell eukaryotes to invertebrates and up to higher mammals.

Based on the FlyBase and Ensembl databases, *fam161* codes for two transcripts with alternative transcription initiation sites but identical coding sequences (RA and RB; figure 1*c*). Fly Fam161 protein has 54% and 49% overall similarity and 22% overall identity with FAM161A and FAM161B isoforms, respectively (figure 1*b*; electronic supplementary material, figures S1 and S2). The FAM161A N-terminal domain mediates interaction with POC1B and POC5 [13]. Fam161 similarity was 64% in the POC1B binding domain and 49% in the POC5 binding domain. The most conserved domain of Fam161 was the N-terminal POC1B binding domain, suggesting a potential interaction between Fam161 and POC1B in flies as well. The least conserved domain of Fam161 was the N-terminal POC5 binding domain, an observation consistent with the absence of POC5 in flies. The most characterized domain in FAM161A was UPF0564, which is found at the centre of the protein’s primary structure and mediates FAM161A dimerization and microtubule binding [20]. Fam161 similarity was 53% in the dimerization domain and 53% in UPF0564. Fam161 similarity was 58% in the C-terminal domain, though the role of the FAM161A C-terminal domain is unknown (electronic supplementary material, figure S1). Like FAM161A and FAM161B, Fam161 is predicted with high confidence by Alphafold to be enriched with α-helical regions [38,39]. This data suggests that Fam161 has a domain organization that is similar to that of its human orthologs.

### 2.2. CRISPR/Cas9-generated fly *fam161* mutants

**Figure 2.**
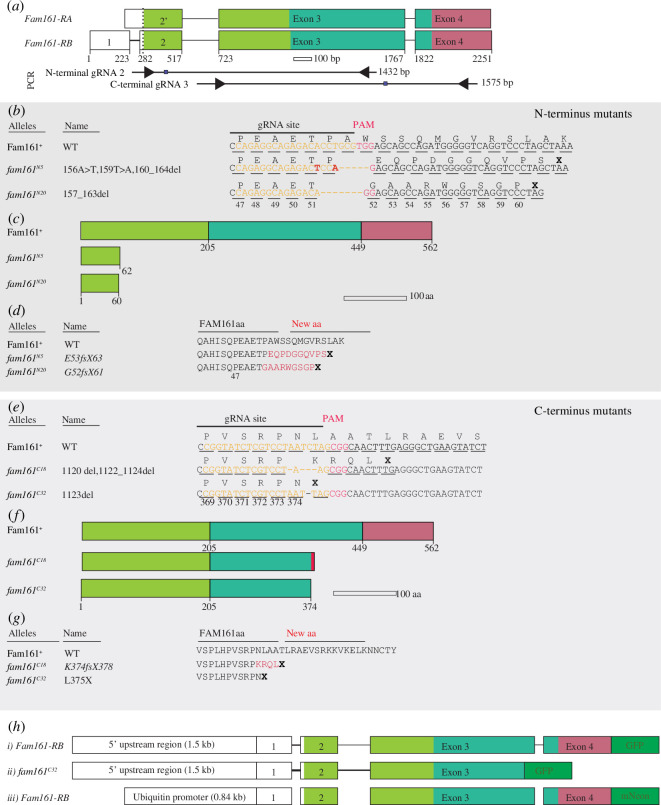
Fam161 (CG1113) mutants generated using CRISPR/Cas9. (*a*) Schematic representation of the fly Fam161 isoform showing four exons and the introns, N-terminal guide RNA 2 binding site, C-terminal guide RNA 3 binding site and screening polymerase chain reaction primer site for mutant analysis. (*b–d*) Fam161 N-terminus mutation description. (*b*) Protein sequence showing the N-terminal gRNA binding site and protospacer adjacent motif (PAM) in wild-type (WT) Fam161 and base substitutions/deletions in the gRNA site of *fam161^N5/20^* mutants. (*c*) Schematic representation of the Fam161 WT protein and *fam161^N5/20^* mutants. (*d*) Figure showing the amino acid sequence of WT Fam161 protein and the change in amino acid sequence and early termination in *fam161^N5/20^* mutants. (*e–g*) Fam161 C-terminus mutation description. (*e*) Protein sequence showing the C-terminal gRNA binding site and PAM in WT Fam161 and insertions or deletions in the gRNA site of *fam161^C18/32^* mutants. (*f*) Schematic representation of the WT Fam161 protein and *fam161^C18/32^* mutants. (*g*) Figure showing the amino acid sequence of WT Fam161 protein and the change in amino acid sequence and early termination in *fam161^C18/32^* mutants. (*h*) Schematic representation of the transgenic fly *fam161* and *fam161^C32^* allele, in which the stop codon is replaced with a Green Fluorescent Protein (GFP)-coding sequence (i) and (ii) mNeon (iii) under the control of the endogenous and ubiquitin promoter, respectively.

To identify Fam161’s essential roles, we generated two types of mutants using the CRISPR/Cas9 method. We designed four guide RNAs: only guide RNA 2, which targeted exon 1 and guide RNA 3, which targeted exon 3 (figure 2*a*), produced insertion/deletion mutations (figure 2; electronic supplementary material, figure S4A) (figure 2*e*; electronic supplementary material, figure S4C).

Exon 1 mutations were at or near amino acid 49, predicting proteins truncated in their N-terminus and missing approximately 500 amino acids (e.g. N-terminus mutants fam161^N5^ and fam161^N20^; figure 2*c*; electronic supplementary material, figure S4B). For example, the fam161^N5^ mutant had two base substitutions and a deletion: an A to T substitution at base 156, a T to A substitution at base 159 and a deletion from base 160 to 164 (figure 2*b*). This mutant has a 5-base deletion, including bases 160 to 164 (figure 2*b*). These base changes resulted in fam161^N5^ having a frameshift from amino acid E53 for 10 amino acids before a stop codon (figure 2*d*). In both mutants, the base deletion was predicted to cause a frameshift, leading to amino acid sequence change before an early stop codon (TAA or TAG; figure 2*b,d*). These two truncated proteins were missing most of the protein (approx. 89%) and were predicted to be severe or complete loss of function alleles.

Exon 3 mutations were near amino acid 374, predicting proteins truncated near their C-terminus and missing approximately 187 amino acids (e.g. C-terminus mutants fam161^C18^ and fam161^C32^; figure 2*f*; electronic supplementary material, figure S4D). For example, fam161^C18^ was missing base 1120 and bases 1122 to 1124 (figure 2*e*) and had a frameshift at amino acid K374, changing four amino acids before an early stop codon (figure 2*g*). Fam161^C32^ was missing base 1123 (figure 2*e*) and had a deletion at amino acid 375 (figure 2*g*). These two truncated proteins were missing approximately 33% of the protein and were predicted to produce a partial loss of function protein.

To study Fam161 localization, we produced three Fam161 transgenes: Fam161GFP and *fam161^C32^* GFP, expressed by the *fam161* promoter and Fam161mNeon, expressed by the ubiquitin promoter (figure 2*h*). All the transgenes are inserted on the second chromosome to promote gene knockdown-rescue experiments.

### 2.3. Fly Fam161 is essential for mating and geotaxis

Mutations in centriole and cilia TZ-specific proteins in flies exhibit a range of phenotypes. The most extreme is a severe loss of function mutation in centriole formation proteins, such as Asterless [40–42], Unc [43,44], Ana2 [45] and Ana1 [46], which results in lethality after emergence from the pupa owing to a complete lack of mechanosensory input needed for proprioception and locomotion. Similarly, severe loss of function mutation in Sas-4 is lethal; the flies develop to the adult stage but die shortly afterwards owing to lack of cilia and flagella [47]. Mutations in Sas-6 are also lethal, with approximately 99% pharate adult mortality rate [48]. Similarly, mutations in SAK lead to immotile sperm and male sterility [49]. More moderate phenotypes are observed in Cep97 mutations that mildly affect centriole structure and include geotaxis defects [50] and mutations in ciliary rootlet protein Rootletin, which lead to impaired climbing and hearing [51]. As a result of our mutations in flies, we expected a mild sensory defect, as a FAM161A mutation in humans and mice causes retinal degeneration [24]. Indeed, Fam161 is dispensable for viability and reproduction in laboratory conditions, and *fam161* homozygotes can reproduce and maintain a culture.

**Figure 3.**
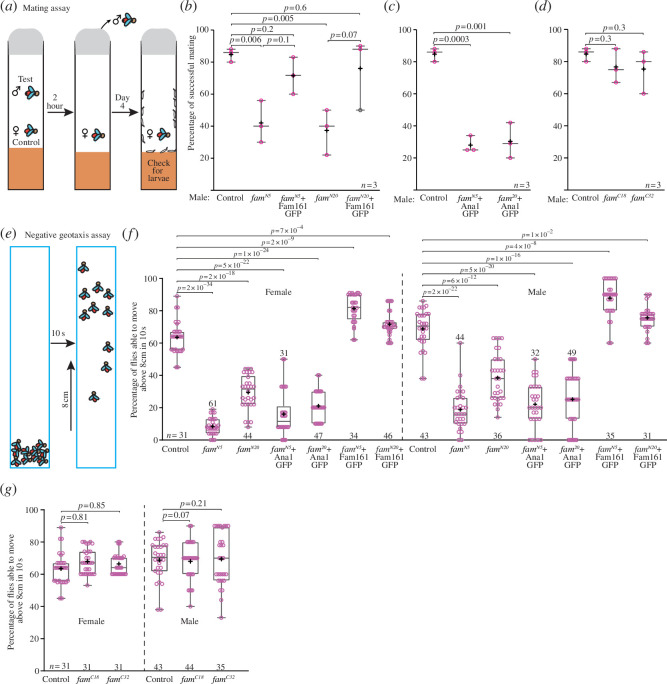
Fam161 is required for mating (*a–d*) and geotaxis (*e–h*). (*a*) Schematic representation of mating assay. (*b*) Mating success comparison between control, N-terminal mutants and rescue flies. *fam161^N5^* and *fam161^N20^* have lower mating success than *w^1118^* control flies. Combining the mutants with Fam161GFP transgene (*fam161^N5^+*Fam161 GFP and *fam161^N20^+*Fam161 GFP) rescues the phenotype. (*c*) Mating success comparison between control and N-terminal mutants with Ana1GFP. As expected, combining the mutants with Ana1GFP transgene (*fam161^N5^+*Ana1 GFP and *fam161^N20^+*Ana1 GFP) failed to rescue the phenotype. (*d*) Mating success comparison between control and C-terminal mutants. *fam161^C18^* and *fam161^C32^* have mating success rates similar to that of control *w^1118^*. (*b–d*) ‘+” indicates the mean. *N* represents the number of independent experiments, with each dot representing the average of 10 matings in a single experiment. (*e*) Schematic representation of geotaxis experiment. (*f*) Climbing ability comparison between control, N-terminal mutants, mutants with Ana1GFP transgene and rescue female and male flies within a given time period. The percentages of *fam161^N5^* and *fam161^N20^* flies that climbed above 8 cm were significantly lower than the *w^1118^* control percentages in both female and male flies. The N-terminal mutants with Fam161GFP transgene (*fam161^N5^+*Fam161 GFP and *fam161^N20^+*Fam161 GFP) have a climbing ability slightly higher than that of *w^1118^*. The mutants with Ana1GFP transgene (*fam161^N5^+*Ana1 GFP and *fam161^N20^+*Ana1 GFP) have comparatively low percentages of flies that were able to climb above 8 cm. (*g*) Climbing ability comparison between control and C-terminal mutants female and male flies within a given time period. The percentages of *fam161^C18^* and *fam161^C32^* flies that could climb above 8 cm are similar to the *w^1118^* control percentages. (*f–h*) ‘+’ indicates the mean. *n* represents the total number of flies used in three independent geotaxis assays.

Fly mating is a complex behaviour requiring the male to have normal proprioception, produce a courtship song and mate [52]. Consequently, mechanosensory defects that cause sensory dysfunction can lead to courtship delay [53]. To test *fam161* homozygote male mating success, we bred them with *w^1118^* control females and calculated the rate of offspring-producing females. We found that the *w^1118^* control males had 85 ± 4% mating success (*n* = 30). The *fam161^C18^* and *fam161^C32^* males had similar rates of success at 77 ± 11% and 75 ± 14%, (*n* = 30, *p* = 0.3 and 0.3; figure 3*d*), suggesting that the Fam161 C-terminus is dispensable for mating. By contrast, the *fam161^N5^* and *fam161^N20^* males had low mating success rates of 42 ± 13% and 37 ± 14% (*n* = 30, *p* = 0.006 and *p* = 0.005; figure 3*b*).

A rescue experiment using the Fam161GFP transgene rescued the mating phenotype of both mutants, resulting in increased mating success rates of 72 ± 16% and 76 ± 22% (*n* = 30), which were rates similar to that of control flies (*p* = 0.2 and *p* = 0.6; figure 3*b*). By contrast, adding a negative control transgene (Ana1GFP) resulted in low mating success rates of 28 ± 5% and 30 ± 11% (*n* = 30, *p* = 0.0003 and *p* = 0.001; figure 3*c*). These results suggest that Fam161, but not its C-terminus, is essential for normal mating.

The embryonic centriole is inherited from the sperm [54]. Mutations in sperm centriole components can lead to abnormal embryonic development and reduced fecundity (the ability to produce many offspring) [12,55,56]. To test if *fam161^N^* affects embryonic development, we bred them and calculated the egg-hatching rate. We found that *w^1118^* control, *fam161^N5^* and *fam161^N20^* had similar hatched egg rates of 50% (72 ± 11%, *n* = 472; 54 ± 11%, *n* = 405, *p* = 0.11; 62 ± 12%, *n* = 503, *p* = 0.34, respectively; electronic supplementary material, figure S5). This observation suggests that maternal and paternal *fam161^N^* were dispensable for embryonic development.

The mating phenotype suggested that Fam161 is essential to the male reproductive process preceding fertilization, e.g. courtship, by possibly affecting sensory functions. Gravity sensation in the fly is mediated by Johnston’s mechanosensory organ, and external and internal mechanosensory organs mediate locomotor coordination [44]. Therefore, to test if fam161 mutant flies have a mechanosensory defect, we performed a negative geotaxis test [57,58]. In this assay, the percentage of flies climbing up an 8 cm wall in 10 s is determined [59]. Compared with control w^1118^, fam161^N5^ and fam161^N20^ flies had a severe climbing defect (males: *p* = 2 × 10^−22^ and *p* = 6 × 10^−12^; females: *p* = 2 × 10^−34^ and *p* = 2 × 10^−18^; figure 3*f*). Furthermore, Fam161GFP transgene (males: *p* = 4 × 10^−8^ and *p* = 1 × 10^−2^; females: *p* = 2 × 10^−9^ and *p* = 7 × 10^−4^), but not Ana1GFP transgene (males: *p* = 5 × 10^−20^ and *p* = 1 × 10^−16^; females: *p* = 5 × 10^−22^ and *p* = 1 × 10^−24^), rescued this geotaxis phenotype (figure 3*f*). The Fam161 mutants with Fam161GFP transgene have a better climbing ability than wild-type control. The C-terminal mutant did not have any climbing defect (figure 3*g*). These results suggest that Fam161, but not its C-terminus, is essential for normal geotaxis.

### 2.4. Fam161 localizes to the sensory neuron centriole and transition zone

**Figure 4.**
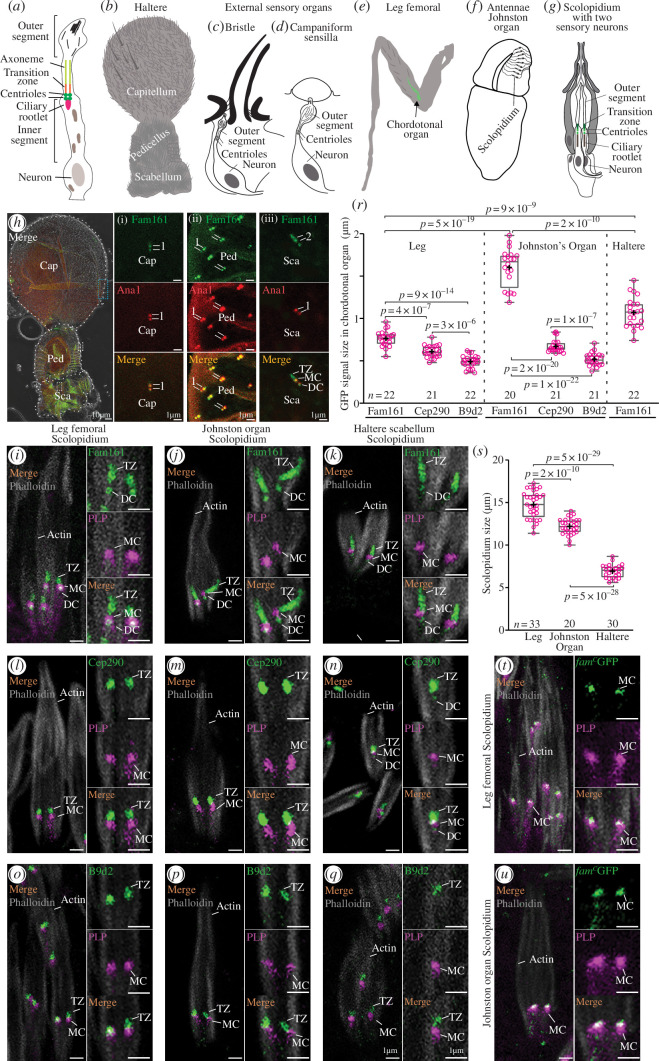
Fam161 localizes to the centriole and TZ in sensory neurons. (*a–f*) Schematic representation of *Drosophila melanogaster* sensory cilium, composed of outer segment, TZ and inner segment (*a*), haltere with three distinct parts (capitellum, pedicellus and scabellum) (*b*), bristle (*c*), campaniform sensilla (*d*), leg femoral showing femoral chordotonal organ (*e*), Johnston’s organ with scolopidium (*f*) and scolopidium (*g*). (*h*) Fam161GFP has arrangement 1 (1) and colocalizes with Ana1tdTomato at the two centrioles in capitellum (i) and pedicellus (ii) and has arrangement 2 (2) in the haltere scabellum (iii), where it labels daughter centriole (DC), mother centriole (MC) and TZ, and colocalizes with Ana1tdTomato at the MC and DC. (*i–q*) Differential localization of Fam161, Cep290 and B9d2 in sensory neurons (*i–k*) Fam161 localizes to the centriole and TZ of the chordotonal organ of leg femoral (*i*) and Johnston’s organ (*j*) and haltere scolopidium (*k*). (*l–n*) Cep290 localizes only to the TZ in the chordotonal organ of the leg femoral (*l*) and Johnston’s organ (*m*) and to the centriole and TZ of the chordotonal organ of haltere scolopidium (*n*). (*o–q*) B9d2 localizes to the TZ of the chordotonal organ of the leg femoral (*o*), Johnston’s organ (*p*) and haltere scolopidium (*q*). (*r*) Quantification showing average Fam161GFP signal length in leg femoral chordotonal organ, Johnston organ and haltere chordotonal organ and Cep290GFP and B9d2GFP length in leg femoral chordotonal organ and Johnston’s organ. (*s*) Quantification showing average scolopidium length in leg femoral chordotonal organ, Johnston’s organ and haltere chordotonal organ. (*t–u*) *Fam161^C32^* localizes above Pericentrin-like-protein (PLP) in the chordotonal organ of leg femoral (*t*) and Johnston’s organ (*u*).

Fly-ciliated mechanosensory neurons (aka type I mechanoreceptor) have a structure similar to the mammalian photoreceptor and *Ca. elegans* sensory neurons, in which the inner segment is connected to the outer segment via a connecting cilium, a long and specialized TZ ([60–62], reviewed in [63,64]; figure 4*a*). Here, we tested Fam161 localization in two categories of fly type I mechanoreceptors:

- external sensory organs found in the haltere, including capitellum bristle sensilla and pedicellus campaniform sensilla (figure 4*b–d*; [65,66]). The haltere is a sensory organ involved with flight stability that is rich in mechanosensory neurons [67,68]; and
- internal sensory organs, also known as chordotonal organs, are found in the haltere scabellum chordotonal organ, leg femoral chordotonal organ and Johnston’s organ (figure 4*e–g*; [68,69]; reviewed in [70]). Chordotonal organs are made of individual sensory units called scolopidia; each has a sensory neuron. Johnston’s organ has many scolopidia (figure 4*f*), and each scolopidia has one or two sensory neurons (figure 4*g*).

Fam161 expression in fly tissues is very low [71,72]. While the low expression is a general property of centriole and ciliary genes in flies [60], compared with other centriolar and transition proteins, FAM161 has very low expression (electronic supplementary material, figure S7). We studied Fam161 localization using the Fam161GFP transgene (figure 4*h*) and the centriolar protein Ana1tdTomato [40]. Fam161 was found to co-localize with Ana1 as two dots in the external organs: in the haltere capitellum (figure 4*h*(i)) and pedicels campaniform sensilla (arrangement 1; figure 4*h*(ii)). This labelling pattern is similar to that observed with other centriolar proteins, such as HYLS1 at the antennae third segment external sensory organs [73] and PACT at external olfactory sensilla [74], suggesting that Fam161 is a centriolar protein in external sensory neurons.

Fam161 also colocalizes with the two Ana1 dots in the haltere scabellum chordotonal organ at the daughter and mother centrioles (figure 4*h*(iii)). However, Fam161 labelling extends beyond the Ana1 staining, suggesting that it also labels the TZ (arrangement 2; figure 4*h*(iii)). This labelling pattern is different from other TZ proteins, such as Cep290, B9d1, Mks1, Cby, Unc, Fam92 and Dzip, which in chordotonal organs are only present in the TZ [4,64]. A similar Fam161 localization in the daughter centriole, mother centriole (basal body) and TZ was observed in the fly leg femoral chordotonal organ (figure 4*i*) and Johnston’s organ scolopidium (figure 4*j*), suggesting that Fam161 is both a centriole and TZ protein in internal sensory neurons. This localization pattern is unique to Fam161, as all other fly centriole/TZ proteins were reported to be localized either to the centriole or TZ of sensory neurons (see discussion).

In the TZ of sensory neurons, Jana *et al*. [4] found a subdivision to a proximal segment that contains most TZ proteins (including B9d1) and a distal TZ segment that contains Cep290 [4]. We found a similar pattern in the TZ of the chordotonal organs of the leg femoral, where B9d2 length was 0.49 ± 0.07 µm, and Cep290 labelling length was 0.61 ± 0.07 µm (*p* = 3 × 10^−6^; figure 4*l,o,r*). Similarly, in Johnston’s organs, B9d2 length was 0.52 ± 0.08 µm, and Cep290 labelling length was 0.67 ± 0.08 µm (*p* = 1 × 10^−7^; figure 4*m,p,r*). These observations confirm the presence of proximal and distal parts of the TZs in the leg femoral chordotonal organ and Johnston’s organ.

Remarkably, Fam161 labelling localized to the TZ is longer than that of Cep290 and B9d2 in the leg femoral chordotonal organ (*p* = 4 × 10^−7^ and 9 × 10^−14^) and Johnston’s organ (*p* = 2 × 10^−20^ and 1 × 10^−22^; figure 4*r*), suggesting another subdivision that we refer to as an extra distal part of the TZ. Furthermore, Fam161 TZ localization further diversifies into varying lengths based on the chordotonal organ location (figure 4*i–k*). Fam161 labelling was the longest in the antenna Johnston organ (1.60 ± 0.23 µm), medium length at the haltere scabellum (1.07 ± 0.20 µm), and shortest in the leg femoral chordotonal organ (0.76 ± 0.09 µm), with statistically significant differences (*p* = 9 × 10^−9^ and 5 × 10^−19^; figure 4*r*).

To gain insight into these length differences, we studied scolopidium length by labelling its actin using tagged phalloidin [75]. We found that the leg femoral scolopidia were longest (14.8 ± 1.6 µm), Johnston’s organ scolopidia were intermediate (12.2 ± 0.9 µm) and haltere scabellum chordotonal organ scolopidia were the shortest (6.9 ± 0.8 µm), with statistically significant differences in the leg and Johnston’s organ (*p* = 5 × 10^−29^ and 5 × 10^−28^; figure 4*s*). These observations indicate that there is no correlation between scolopidium size and Fam161 TZ localization length.

Overall, these data suggest that Fam161 localization has two distinct arrangements: one labelling the two centrioles in external sensory organs and the other labelling the two centrioles and TZ in internal/chordotonal sensory organs. In the TZ of internal sensory neurons, Fam161 has diverse localization patterns that extend beyond the proximal and distal TZ, marking a novel compartment, the extra distal part of the TZ.

To get insight into the ability of the C-terminus mutation to exhibit wild-type negative geotaxis and mating assays, we studied its localization using a tracked transgene mimicking the mutation in fam161c32 (figure 3*h*). We found that the truncated protein localizes distal to the PLP labelling in the fly leg femoral chordotonal organ (figure 4*t*) and Johnston’s organ scolopidium (figure 4*u*), suggesting that the C-terminus Fam161 mutant is at least partially functional and can target to the centriole/TZ of sensory neurons. This localization pattern was similar to Poc1A and Poc1B localization in the leg (electronic supplementary material, figure S6-Aiv-v) and Johnston’s organ (electronic supplementary material, figure S6-Civ-v) chordotonal organ. Since mouse POC1B interacts with the N-terminus of FAM161A [13], it is possible that the partial localization of fam161c32 was owing to maintaining the intact N-terminal domain.

### 2.5. Overexpressed Fam161 localizes to sperm centrioles and the transition zone during spermatogenesis

In spermatocytes, centrioles elongate to form a pair of giant centrioles, each forming a cilium-like structure containing a long TZ (approx. 1 µm; [76–78]). After meiosis, the spermatid inherits one of these giant centrioles, the axoneme elongates to form the sperm tail and the TZ migrates to the axoneme tip [79,80]. Near the giant centriole, an atypical centriole, the proximal centriole-like (PCL), is formed [46]. Finally, the sperm centriole proteins are remodelled during spermatogenesis, during which most centriolar proteins are eliminated and become undetectable in the spermatozoan giant centriole and PCL [12].

**Figure 5.**
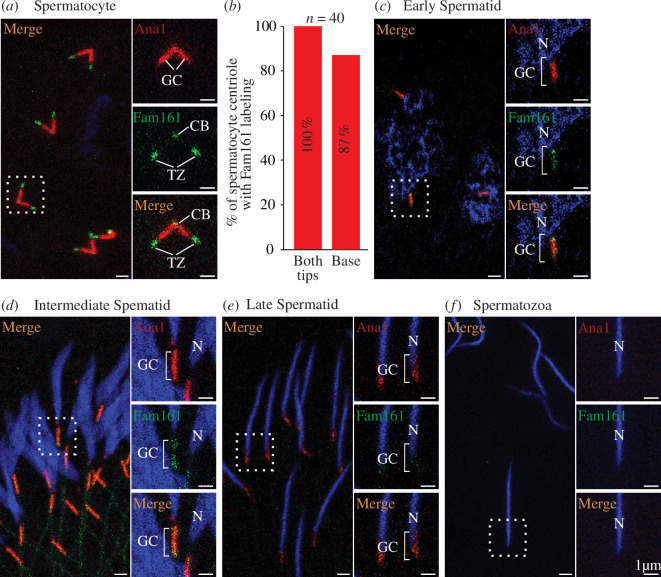
Overexpressed Fam161 localizes to sperm centrioles and the TZ during spermatogenesis. (*a*) Fam161Neon labels the tip and base of the spermatocyte centriole. (*b*) Quantification showing the percentage of centrioles with Fam161Neon labelling the tip and base of the spermatocyte centriole. (*c,d*) Fam161Neon colocalizes with Ana1tdTomato in the giant centriole of early and intermediate spermatids, with Fam161Neon being concentrated at the tip. (*e*) Ana1tdTomato continues to label the giant centriole during the late spermatid stage, whereas Fam161neon is barely present. (*f*) Ana1tdTomato and Fam161Neon are both stripped from the mature sperm centriole during spermiogenesis.

We did not detect Fam161GFP expressed by its promoter in the testis. Therefore, we studied the localization of overexpressed Fam161 during spermatogenesis using flies containing the Fam161mNeon and Ana1tdTomato transgenes. Spermatogenesis occurs in spatiotemporal order in the testis, during which sperm cell stages can be identified by their location and the shape of the sperm nucleus and centriole [81,82]. We found that Fam161 localizes to the centriole base (87%) and the TZ (100%) at the spermatocyte stage (figure 5*a,b*) and to the centriole base during the round (figure 5*c*), intermediate (figure 5*d*) and late (figure 5*e*) spermatid stages. Like most centriolar proteins, Fam161 is undetected in mature spermatozoa (figure 5*f*). These findings suggest that during spermatogenesis, over-expressed Fam161 can localize to the sperm centriole and TZ in a stage-specific manner. The localization pattern of Fam161 was different from that of other transition proteins, such as Cep290, Mks1 and B9d2, which are present only in the TZ [79], or centriolar proteins Sas4, Sas6 and Ana1, which are found only in the centrioles [4].

## 3. Discussion

Centrioles are abundant and essential cellular structures, yet mutating any one of the centriolar proteins leads to a distinct and specific phenotype. For example, unlike many other centriolar proteins that cause lethality, FAM161A mutation results in blindness in mammals. One explanation for this phenomenon is that the centriole plays a slightly different role in different cell types. To gain insight into these differences, we studied fly Fam161 localization and its effects in various cells. Here, we discovered that fly protein Fam161 is an essential centriole and TZ protein that exhibits cell-type specific localization patterns. Further comparison of the localization patterns of various centrioles and TZ proteins in various sensory neurons of fruit flies, based on previously published data, revealed that Fam161 is the centriolar protein that displays the most diverse localization pattern.

As Fam161 has diverse localization patterns in sensory cilia, we wondered if other centriolar and TZ proteins have similar localization patterns. To investigate that, we reviewed the literature on fly centriolar and TZ proteins and studied some of them in the chordotonal organ of the leg femoral, antenna Johnston’s organ and haltere scolopidium.

**Figure 6.**
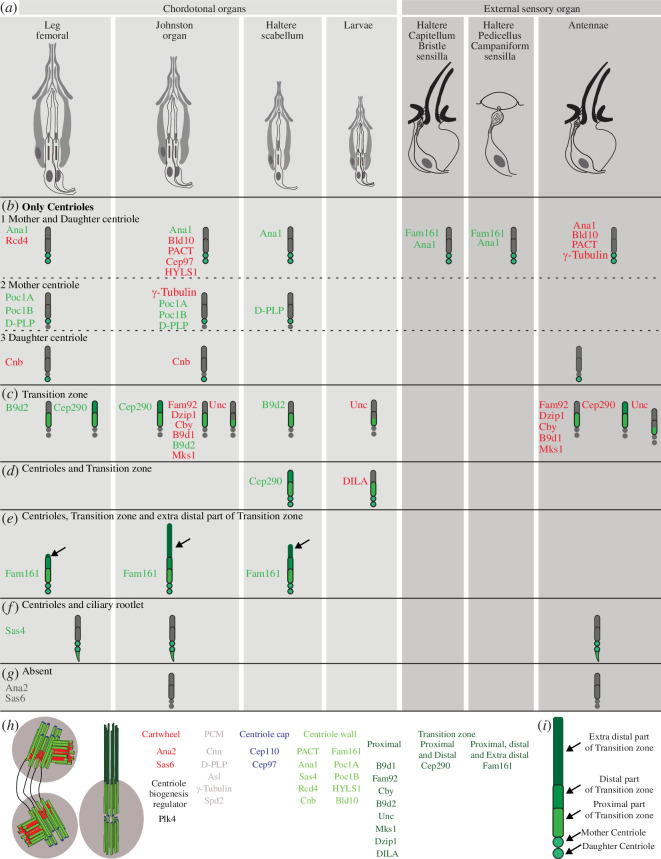
Summary of centriole and TZ protein localization in fly ciliated cells. (*a*) Ciliated sensory neuron types are depicted on the top. (*b–g*) Four main patterns of localization were found. (*b-1*) Ana1 and PACT are proteins that are localized specifically and consistently to mother and daughter centrioles. This group includes seven proteins. (*b-2*) Poc1A and Poc1B are proteins that are localized specifically and consistently to mother centrioles. This group includes three proteins. (*b-3*) Cnb is a protein that is localized specifically and consistently to the daughter centriole. This group includes one protein. (*c*) B9d1, B9d2 and Mks1 are proteins that localize specifically and consistently to the TZ. This group includes eight proteins. (*d*) Cep290 and DILA are proteins that localize to mother and daughter centrioles as well as the TZ in a cell type-specific manner. (*e*) Fam161 localizes to centrioles and labels the TZ at varying lengths. (*f*) Sas4 localizes to the mother and daughter centrioles and ciliary rootlet. (*g*) Sas6 and Ana2 are proteins that are consistently absent in the centrioles or TZ. Plk4 and CP110 had no available study in sensory neurons. (*h*) Functional types of centriole and TZ proteins based on the literature. (*i*) Model of the TZ compartments: proximal, distal and extra distal. References for fly protein localization: HYLS1, Rcd4 [73,83]; Cep97 [50]; Cnb [74,83]; Fam92, Cby and Dzip1 [64]; PACT [74]; Unc and DILA [84]; Unc, Chibby, B9d1, Mks1, and Cep290 [4]; Bld10 [4]; Ana2 and Sas−6 [4]; Asl [40]; Bld10 [59]. The proteins with green-coloured text were analysed in this study, while those with red-coloured text were obtained from published data.

We found five patterns and some sub-patterns of localization in sensory cilia (figure 6):

- proteins that are found only in the centrioles:
  - proteins that are localized specifically and consistently to both the mother and daughter centrioles. This subgroup includes six proteins: Ana1 [74,85], PACT [74], Rcd4 [73], HYLS1 [73], Bld10 [4] and Cep97 [50];
  - proteins that are localized only to the mother centrioles. This subgroup includes four proteins: Poc1A, Poc1B, γ-tubulin [4] and PLP [83]; and
  - proteins that are localized only to the daughter centrioles. This subgroup includes one protein: Cnb [74,83];
- proteins that are found only in the TZ. This group includes seven proteins: B9d1 [4], B9d2, Mks1 [4], Fam92 [64], Dzip1 [64], Cby [4,64] and Unc [4,84];
- proteins that are found in both the centrioles and the TZ. This group includes two proteins: Cep290 and DILA. Cep290 is always found in the TZ [4], but it is also found in both centrioles of haltere scolopidia, while DILA is found in both the centriole and the TZ of larvae chordotonal organs [84];
- proteins that are found in both centrioles and the TZ, with varying lengths based on location. This group includes Fam161, which is always found in both centrioles but sometimes in the TZ at varying lengths;
- proteins that are found in both centrioles and ciliary rootlets. This group includes one protein: Sas4 [4,86]; and
- proteins of unknown localization, which include Plk4 and CP110.

Fam161 localizes to the centrioles in the external sensory organs of the capitellum and pedicellus of the haltere. Additionally, it localizes to the centriole and TZ, labelling the cilia at varying lengths in the chordotonal organ of the haltere scabellum, leg and Johnston’s organ. We also observed that Cep290 is present in the centriole of the chordotonal organ of the haltere scabellum (figure 4*n*). The differential localization of Fam161 and Cep290 in different chordotonal organs suggests a diversity in ciliary base protein composition between them. The diversity in ciliary base protein composition among various cilium types indicates that specific fly mechanosensory cilia may serve as models for studying centriole and TZ protein adaptations.

Notably, a mutation in the fly *fam161* gene leads to abnormal geotaxis and affects mating behaviour. The Fam161 mutation does not affect localization in the chordotonal organ. This observation suggests that Fam161 might have various interacting partners based on its localization or that there is a compensatory mechanism that explains the relatively mild overall effect of the Fam161 mutation in flies.

FAM161A interacts with multiple centriolar and TZ proteins, such as the centriolar proteins POC1B, POC5 and OFD1, the centrosomal protein Lebercilin, SDCCAG8 and the TZ protein CEP290 [13,20,87,88]. When overexpressed in cells, FAM161A localizes to both centrioles, cytoplasmic microtubules and cytoplasmic aggregates [13]. The centriolar localization has been reproduced *in vivo* and represents its physiological connection [89]; the cytoplasmic microtubule localization results from the affinity of FAM161A for microtubules [89] and the cytoplasmic aggregate localization results from the direct interaction of FAM161A with mislocalized centriolar proteins like POC1B, POC5 and centrin [87]. These cytoplasmic localizations are a sort of non-physiological overexpression. The mechanism of FAM161A-specific localization to the centriole and TZ is an important future aim.

FAM161A is a component of the centriole and TZ inner scaffold structure in human and mouse cells [18,27]. The centriole inner scaffold structure is present in single-cell eukaryotes such as *Paramecium tetraurelia* and *Ch. reinhardtii* [18], and FAM161A is conserved in these species and may be one of its components. By contrast, in mammalian photoreceptors, FAM161A was also found, in addition to the centriole, in the long TZ known as the connecting cilium [19,90]. Similarly, the *Ca. elegans* FAM161A orthologue, FAM-161, is a centriole and TZ protein of sensory ciliary neurons [16], suggesting that FAM161A evolved a new function in the animal sensory neuron long TZ. We propose that centriole luminal scaffolds first evolved as centriolar proteins and later evolved a scaffolding role in specialized long TZs commonly found in sensory neurons of animals.

## 4. Methods

### 4.1. Flies

We used the centriolar and TZ transgenic flies Ana1tdTomato [40], Ana1-GFP [46], Sas−4GFP [46], Poc1AGFP, Poc1ABGFP [12], Cep290GFP and B9d2GFP [79].

*Drosophila* Fam161GFP reporter was generated by cloning the 1.522 kb sequence upstream of the *fam161* ATG of isoform CG1113-RB up to the last codon before the stop codon. This sequence includes promoters, exons and introns for a total size of 3.467 kb. This sequence was placed immediately adjacent to the predicted initiator methionine (ATG) up to the second to the last codon between *Pac*I and *Not*I sites into the p{UAST} GFP vector. Similarly, Fam161mNeon was generated by cloning the coding sequence of isoform CG1113-RB between *Asc*I and *Not*I in p{UAST}-Ubiquitin-mNeon vector. *P*-element-mediated germline transformations were performed by BestGene (Chino Hills, CA). Information about polymerase chain reaction (PCR) primers used for amplification of the *fam161* gene and coding sequence is in the electronic supplementary material, tables S1 and S2**,** respectively. Sequencing primers used for cloning confirmation in the p{UAST} GFP and p{UAST}-Ubiquitin-mNeon vector are in the electronic supplementary material, table S3.

Fam161GFP/Ana1tdTomato flies were generated by crossing Fam161GFP flies with Ana1tdTomato transgenic flies. Fam161-mNEON/Ana1tdTomato flies were generated using a similar approach.

To create knockout mutants using CRISPR/Cas9, guide RNAs targeting the N- and C-terminus of Fam161 were designed using the FlyCRISPR online tool (flyCRISPR.molbio.wisc.edu). The target sites were CAGAGGCAGAGACACCTGCG and GGTATCTCGTCCTAATCTAG for the N- and C-terminus, respectively, of the *fam161* gene. The sequences of the forward and reverse oligonucleotides for gRNA synthesis are in the electronic supplementary material, table S4. The forward and reverse oligos for guide RNA were designed such that they have a sticky end for the digested pCFD5 vector. The forward and reverse oligos were ordered from Integrated DNA Technologies.

The forward and reverse oligos for guide RNA synthesis were re-suspended in sterile, double-distilled water (ddH2O) to make a 100 μM stock solution. To prepare the annealed oligos, 1 μl top oligo (100 μM stock), 1 μl bottom oligo (100 μM stock), 1 μl 10-fold T4 ligation buffer (NEB) and 7 μl ddH2O were added to a 1.5 ml microcentrifuge tube. The 1.5 ml microcentrifuge tube was then placed in a 95°C hot block for 5 min, after which it was allowed a couple of hours to cool to room temperature. The annealed oligos were then cloned into the pCFD5 vector. The pCFD5 plasmid was digested with the Bbs1 restriction enzyme (New England Biolabs). 8 μg of plasmid DNA were digested with 1 μl enzyme (10 units) and 3 μl 10-fold buffer in a 30 μl reaction for 2−4 h at 37°C. The digested plasmid was run on a 1% agarose gel. The linear backbone was cut and purified using a Qiagen gel purification kit. Purified DNA was eluted in 25 μl sterile ddH2O, and DNA concentration was measured. The annealed oligos were cloned in a digested pCFD5 vector. The appropriate concentrations of the linear vector and oligos were determined using the NEBioCalculator.

The ligation reaction was set with: X μl *BbsI* digested pCFD5 (60 ng), 1 μl annealed oligos diluted 1 : 200 in ddH_2_O, 1.5 μl 10× T4 ligation buffer (NEB), X μl ddH_2_O, 1 μl T4 DNA Ligase (NEB), for a total volume of 15 μl. The ligation mixture was incubated at 16°C overnight. Two microlitres of the ligation reaction were used to transform 50 μl of chemically competent bacteria. Seven hundred microlitres of Luria–Bertani (LB) was added to the transformation mixture, and the cells were allowed to recover for 45 min in a rotating 37°C incubator. After 45 min, 100 μl of the transformation mixture were plated on ampicillin plates. The plates were incubated at 37°C overnight. On the following day, single colonies were picked and inoculated in LB medium overnight. On the following day, the plasmid was isolated using a Qiagen Mini prep kit.

The isolated plasmid was sent for sequencing using the sequencing primer listed in the electronic supplementary material, table S5. The cloned plasmid with gRNA was sent to BestGene. P-element-mediated germline transformations were performed by BestGene (Chino Hills, CA). To generate Fam161 mutations, the gRNA flies were crossed with Cas9 flies (Bloomington stock number 66554). We generated flies with Cas9 on the X chromosome and guide RNA on the second chromosome and expected these flies to have a mutation in the *fam161* gene on the third chromosome (crossing set-up explained in the electronic supplementary material, figure S3). We set up more than 50 different crosses. Genomic DNA was isolated from single flies from each cross.

Mutant flies expressing GFP fusion proteins were generated by crossing mutant flies with Ana1GFP, Sas−4GFP, Poc1AGFP, Poc1ABGFP, Cep290GFP and B9d2GFP transgenic flies, such that the respective GFP transgene was on the second chromosome and *fam161* mutation was on the third chromosome.

Rescue flies were generated by crossing mutant flies with Fam161GFP flies. The rescue flies have Fam161GFP on the second chromosome and *fam161* mutation on the third chromosome.

A single fly was used for genomic DNA isolation. A single fly was kept in a 1.5 ml microcentrifuge tube and stored at −20°C for 10 min. After 10 min, homogenization buffer (50 mM Ethylenediaminetetraacetic acid (EDTA) and 100 mM Tris) was added to the microcentrifuge tube, and the fly was crushed in the homogenization buffer. One microlitre of proteinase K and one-tenth volume of 10% sodium dodecyl sulfate (SDS) were added to the microcentrifuge tube, and the solution was mixed by vortexing and spinning down for a few seconds.

The microcentrifuge tube was kept in the heat block at 65°C for 1 h. After 1 h, a volume of phenol equal to that of the solution was added to the tube and vortexed at high speed for 5 min. After vortexing, the tube was centrifuged at 15 000 rpm for 5 min. After centrifugation, the supernatant was carefully removed and transferred to a new microcentrifuge tube. An equal volume of chloroform (isoamyl alcohol (24 : 1)) was added to the microcentrifuge tube, and the tube was vortexed at 15 000 rpm for 5 min. After centrifugation, again, the supernatant was gently pipetted out and transferred to a new tube. One-tenth volume of sodium acetate and twice the volume of 100% ethanol was added to the tube, and the tube was left at room temperature for 1 h. After that, the tube was centrifuged at 15 000 rpm for 10 min. The supernatant was then carefully removed and discarded without disturbing the pellet, and 500 μl of 70% ethanol was added and centrifuged for 5 min. After centrifugation, the supernatant was discarded, the tube was allowed to air dry for a few minutes, and the pellet was dissolved in 20 μl Tris-EDTA (TE) buffer.

After genomic DNA isolation, PCR was done to amplify the approximately 1 kb region of the *fam161* gene. The primers were designed such that the forward and reverse primers were approximately 420 bp upstream and downstream of the guide RNA site. Information about the primers is in the electronic supplementary material, table S6. PCR was performed using Q5 polymerase from NEB. For this, 1.25 μl of forward and reverse primers, 0.5 μl of dNTPs, 100 ng of genomic DNA, 5 μl of reaction buffer, 5 μl of GC enhancer and 0.25 μl of Q5 DNA polymerase were added together along with nuclease-free water such that the final PCR mixture volume was 25 μl. The PCR cycle was used, as instructed by NEB. After completion of the PCR cycle, the PCR product was run in 1% agarose gel under 100 V for 45 min to 1 h. Then, the gel was viewed under UV light, and the required band was cut from the gel and purified using a Qiagen gel purification kit. The purified product was sent for sequencing to Genewiz using the sequencing primer listed in the electronic supplementary material, table S7.

### 4.2. Negative geotaxis assay

For the negative geotaxis assay, *w^1118^* flies were used as a control. Each negative geotaxis assay was performed using 10 male or female flies that were placed in a 20 cm-long hollow tube. Unless indicated otherwise, all flies were aged 4 days. The flies were kept in the 20 cm-long hollow tube for at least an hour before experimenting. The flies were tapped down to the bottom of the tube, and the number of flies able to climb the wall above 8 cm within 10 s was counted. This process was repeated 10 times, with a 1 min interval between each session. The same procedure was repeated for other flies as well. Protocol was used as stated in [59].

### 4.3. Mating assay

For the mating assay, 4-day-old *w^1118^* virgin females were crossed with 4-day-old mutant males, and a cross between a *w^1118^* male and a *w^1118^* female was set up as control. All crosses were set up between single males and single females. For a fly with a particular genotype, 10 different crosses were set. The vials were incubated at 25°C for about 4 h. The male flies were discarded after 4 h, and the vials were incubated at 25°C for 4 days. On day 4, females were discarded, and each vial was carefully inspected; the presence of larvae indicated successful mating. Then, the percentage of successful mating was calculated for each mutant and controls. This experiment was repeated at least three times.

### 4.4. Hatching assay

For this assay, 4-day-old male flies were crossed with 4-day-old virgin female flies of the same genotype for 12 h in a vial at 25°C. All crosses were set up between single males and single females. *w^1118^* flies were used as a control. After 12 h, the male flies were discarded, and the females were transferred to a Petri dish with grapefruit agar (Genessesci). Active yeast paste was smeared in the middle of the grapefruit agar to induce females to lay eggs. The female flies were kept in the grapefruit agar for 4 days at 25°C and discarded after day 4. Then, the number of eggs in each agar was counted. After day 10, the number of eggs hatched into larvae was counted, and the percentage of successful hatching was calculated for each mutant and control fly. This experiment was repeated at least three times.

### 4.5. Fluorescent microscopy

For imaging of the haltere, Fam161GFP and Fam161GFP/Ana1tdTomato fly pupae were used. The haltere was plucked using forceps, kept on a glass slide, squashed with a coverslip and viewed under a confocal SP8 microscope.

For staining the scolopidium, the leg and Johnston’s organ of the pupa were dissected in phosphate-buffered saline (PBS) buffer, kept on a slide and squashed with a coverslip. The slides were incubated in a 37°C incubator for at least 10 min to properly glue the leg and Johnston’s organ to the glass slides. Then, the slides were snap frozen in liquid nitrogen for 5 min, the coverslip was removed using forceps, and the slides were placed in a slotted Coplin jar with ice-cold methanol for 5 min. Then, the slides were washed with PBS for 1 min, permeabilized using 0.1% phosphate-buffered saline with Triton x-100 (PBST) for 20 min, and blocked with 1% phosphate-buffered saline with Triton x-100 and Bovine serum albumin (PBSTB) for 30 min. After blocking, a primary antibody was added (GFP, 1 : 100; PLP, 1 : 100) and incubated at 4°C overnight. On the following day, the slides were washed three times for 5 min with PBST in a slotted Coplin jar. After that, secondary antibodies (chicken anti-goat Alexa 488, 1 : 400; donkey anti-rabbit Alexa 647, 1 : 400) and Phalloidin TRITC (1 : 200) were added and incubated at room temperature for 1 h. After secondary antibody incubation, the slides were washed thrice for 5 min with PBST and thrice for 5 min with PBS in a slotted Coplin jar. After that, excess PBS was wiped using Kim wipes, and a mounting reagent with 4',6-diamidino-2-phenylindole (DAPI) was added over the sample; a coverslip was placed and sealed using nail polish. Slides were stored at −20°C until used for imaging.

For imaging the testes of Fam161mNeon/Ana1tdTomato flies, testes of adult flies were dissected in PBS, placed on a glass slide, squashed with a coverslip and snap frozen in liquid nitrogen for 5 min. The coverslip was then removed, and the slide was washed with PBS. Mounting reagent with DAPI was then added over the sample, a coverslip was placed and sealed with nail polish and the samples were imaged immediately using an SP8 confocal microscope.

### 4.6. Antibodies used

We used primary goat GFP antibody from Rockland Immunochemicals (item number 600-101-215), PLP from Dr Jordan W. Raff, and Rhodamine phalloidin from Fisher Scientific (catalogue number 50646256).

### 4.7. Confocal microscopy

Slides were imaged using a Leica SP8 confocal microscope in brightR mode for regular imaging and photon counting mode for quantification using 630× magnification, 1.5×, 3× or 6× zoom and 2048 × 2048, 1024 × 1024 or 512 × 512-pixel format. Scan times were 400 Hz, and line average, line accuracy and frame average were 1, with a frame accuracy of 2 per scan. We used two, three or four sequences depending on the immunostaining. For imaging the scolopidium of the chordotonal organs, we used three sequences: sequence 1 activated a 488 nm laser to collect the Alexa Flour 488 dye signal, sequence 2 activated a 561 nm laser to collect the phalloidin signal that stains the actin of scolopale rods and sequence 3 activated a 633 nm laser to capture Alexa Fluor 647 dye signals. All lasers were set at 4% power.

For imaging tests, we used three sequences. Sequence 1 captured DNA staining via DAPI; for this, we activated a 410 nm (UV) laser at 0.1% power. Sequence 2 activated a 488 nm laser to collect the GFP or Alexa Flour 488 dye signal and sequence 3 activated a 633 nm laser to capture Alexa Fluor 647 dye or a 561 nm laser to capture the tdTomato signal, depending upon the staining. Lasers for sequences 2 and 3 were set at 4% power.

We collected multiple (10–20), 0.3 μm-thick Z sections from the top to the bottom of the haltere, testes, leg femoral chordotonal organ and Johnston’s organ. We generated a maximum projection of all the data collected using the LASX program. Images were captured with a gain of 100.

### 4.8. Statistics

Graphs were plotted using GraphPad Prism. A *t*‐test was performed using the T.TEST function in Excel.

## Data Availability

Further information and requests for resources and reagents should be directed to T.A.-R., corresponding author. All experimental data are contained in the paper and the electronic supplementary material [91].
